# COVID-19 Symptoms and Duration of Rapid Antigen Test Positivity at a Community Testing and Surveillance Site During Pre-Delta, Delta, and Omicron BA.1 Periods

**DOI:** 10.1001/jamanetworkopen.2022.35844

**Published:** 2022-10-10

**Authors:** Carina Marquez, Andrew D. Kerkhoff, John Schrom, Susana Rojas, Douglas Black, Anthea Mitchell, Chung-Yu Wang, Genay Pilarowski, Salustiano Ribeiro, Diane Jones, Joselin Payan, Simone Manganelli, Susy Rojas, Jonathan Lemus, Vivek Jain, Gabriel Chamie, Valerie Tulier-Laiwa, Maya Petersen, Joseph DeRisi, Diane V. Havlir

**Affiliations:** 1 Division of HIV, Infectious Diseases and Global Medicine, Zuckerberg San Francisco General Hospital, University of California, San Francisco, San Francisco; 2 The San Francisco Latino Task Force-Response to COVID-19, San Francisco, California; 3 Chan-Zuckerberg Biohub, San Francisco, California; 4 Unidos en Salud, San Francisco, California; 5 Bay Area Phlebotomy and Laboratory Services, San Francisco, California; 6 Division of Biostatistics, School of Public Health, University of California, Berkeley, Berkeley

## Abstract

**Importance:**

Characterizing the clinical symptoms and evolution of community-based SARS-CoV-2 infections may inform health practitioners and public health officials in a rapidly changing landscape of population immunity and viral variants.

**Objectives:**

To compare COVID-19 symptoms among people testing positive with a rapid antigen test (RAT) during the Omicron BA.1 variant period (December 1, 2021, to January 30, 2022) with the pre-Delta (January 10 to May 31, 2021) and Delta (June 1 to November 30, 2021) variant periods and to assess the duration of RAT positivity during the Omicron BA.1 surge.

**Design, Setting, and Participants:**

This cross-sectional study was conducted from January 10, 2021, to January 31, 2022, at a walk-up community COVID-19 testing site in San Francisco, California. Participants included children and adults seeking COVID-19 testing with an RAT, regardless of age, vaccine status, or symptoms.

**Main Outcomes and Measures:**

Fisher exact tests or χ^2^ tests were used to compare COVID-19 symptoms during the Omicron BA.1 period with the pre-Delta and Delta periods for vaccination status and age group. Among people returning for repeated testing during the Omicron period, the proportion with a positive RAT between 4 and 14 days from symptom onset or since first positive test if asymptomatic was estimated.

**Results:**

Among 63 277 persons tested (median [IQR] age, 32 [21-44] years, with 12.0% younger than 12 years; 52.0% women; and 68.5% Latinx), a total of 18 301 people (28.9%) reported symptoms, of whom 4565 (24.9%) tested positive for COVID-19. During the Omicron BA.1 period, 3032 of 7283 symptomatic participants (41.6%) tested positive, and the numbers of these reporting cough and sore throat were higher than during pre-Delta and Delta periods (cough: 2044 [67.4%] vs 546 [51.3%] of 1065 participants, *P* < .001 for pre-Delta, and 281 [60.0%] of 468 participants, *P* = .002, for Delta; sore throat: 1316 [43.4%] vs 315 [29.6%] of 1065 participants, *P* < .001 for pre-Delta, and 136 [29.1%] of 468 participants, *P* < .001, for Delta). Compared with the 1065 patients with positive test results in the pre-Delta period, congestion among the 3032 with positive results during the Omicron BA.1 period was more common (1177 [38.8%] vs 294 [27.6%] participants, *P* < .001), and loss of taste or smell (160 [5.3%] vs 183 [17.2%] participants, *P* < .001) and fever (921 [30.4%] vs 369 [34.7%] participants, *P* = .01) were less common. In addition, during the Omicron BA.1 period, fever was less common among the people with positive test results who had received a vaccine booster compared with those with positive test results who were unvaccinated (97 [22.5%] of 432 vs 42 [36.2%] of 116 participants, *P* = .003), and fever and myalgia were less common among participants who had received a booster compared with those with positive results who had received only a primary series (fever: 97 [22.5%] of 432 vs 559 [32.8%] of 1705 participants, *P* < .001; myalgia: 115 [26.6%] of 432 vs 580 [34.0%] of 1705 participants, *P* = .003). During the Omicron BA.1 period, 5 days after symptom onset, 507 of 1613 people (31.1%) with COVID-19 stated that their symptoms were similar, and 95 people (5.9%) reported worsening symptoms. Among people testing positive, 80.2% of participants who were symptomatic and retested remained positive 5 days after symptom onset.

**Conclusions and Relevance:**

In this cross-sectional study, COVID-19 upper respiratory tract symptoms were more commonly reported during the Omicron BA.1 period than during the pre-Delta and Delta periods, with differences by vaccination status. Rapid antigen test positivity remained high 5 days after symptom onset, supporting guidelines requiring a negative test to inform the length of the isolation period.

## Key Points

**Questions:**

During the Omicron BA.1 period, were there differences with the pre-Delta and Delta periods in reported COVID-19 symptoms, and what was the duration of rapid antigen test positivity during the Omicron BA.1 period?

**Findings:**

In this cross-sectional study of 63 277 participants conducted at a walk-up community testing site, patients more commonly reported COVID-19 upper respiratory tract symptoms during the Omicron BA.1 period than the pre-Delta and Delta periods, with differences by vaccination status and age. During the Omicron BA.1 period, 5 days after symptom onset, 80% of participants remained positive via a rapid antigen test.

**Meaning:**

These findings indicate differences in symptoms in the BA.1 Omicron period vs the pre-Delta and Delta periods, which may be associated with rising population immunity as well as different SARS-CoV-2 variants, and positivity remained high 5 days after symptom onset in the BA.1 Omicron period.

## Teaser

This cross-sectional study compares COVID-19 symptoms during the Omicron BA.1 period with the pre-Delta and Delta variant periods and assesses the duration of rapid antigen test positivity at a walk-up community testing site.

## Introduction

Symptoms of COVID-19 are an important entry point into testing, treatment, and isolation. Prompt entry into this sequence of care is crucial to breaking the chains of transmission and starting antiviral treatment early in infection.^1,2,3^ As population immunity and viral variants evolve in the ongoing SARS CoV-2 pandemic, understanding COVID-19 symptoms, their duration, and the use of changing diagnostic modalities, such as rapid antigen tests (RATs), can help inform medical professionals and public health leaders about clinical management and key policy questions. National studies from the UK have demonstrated changes in COVID-19 symptom profile by viral variant.^4,5,6^ However, to our knowledge, there has not been a large study of symptom variation by variant period among outpatients outside the UK. Furthermore, there have been relatively few data during the Omicron BA.1 surge on COVID-19 symptoms, their duration, variation in adult and pediatric populations, and variation by vaccination status.

Understanding the duration of symptoms and infectiousness can inform many policy questions, including return to work guidelines, and individual decisions during COVID-19 surges. Although the US Centers for Disease Control and Prevention recommended return to work after 5 days if symptoms improve,^7^ a recent study from Alaska^8^ and 2 studies^9,10^ of health care workers showed that more than 50% to 80% of people remain RAT-positive 5 days after symptom onset. Those data raise concerns about persistent infectiousness after 5 days because antigen test positivity strongly correlates with the presence of viable virus.^11,12,13,14,15^

This study assessed persons testing at a walk-up COVID-19 testing site in San Francisco that serves Latinx residents^16^—a community disproportionately affected by COVID-19^16^—to first determine the prevalence and characteristics of specific symptoms among symptomatic COVID-19–positive persons during the Omicron period compared with the Delta and pre-Delta periods as population immunity and variants evolved. We further sought to assess how symptoms varied by vaccination status and age and to characterize symptom duration and persistence of a positive RAT during the Omicron BA.1 period.

## Methods

### Study Design, Setting, and Participants

This cross-sectional study was conducted between January 10, 2021, and January 31, 2022, at the Unidos en Salud neighborhood testing and vaccine site, which is located in the Mission District of San Francisco, California. The University of California, San Francisco Committee on Human Research determined that the study was exempt from review because it met criteria for public health surveillance. All participants provided written informed consent in their preferred language prior to survey administration and COVID-19 testing. No one received compensation or was offered any incentive for participating in this study.

The Unidos en Salud neighborhood testing and vaccination site was codesigned through a community-academic partnership among the San Francisco Latino Task Force COVID Response, University of California, San Francisco, and the Chan Zuckerberg Biohub.^2,17^ The site serves predominantly low-income Latinx persons, a large majority of whom are essential workers with low wages.^3^ It is conveniently located near a busy transportation hub and offers free walk-up testing with no request for residency or health insurance. Throughout the study period, we consistently conducted community outreach, with messages stressing the importance of regular testing regardless of symptoms and vaccination status owing to the elevated risk of acquiring COVID-19 in the population served by the site. This report followed the Strengthening the Reporting of Observational Studies in Epidemiology (STROBE) guideline for reporting cross-sectional studies.

### Procedures and Samples

All participants or their caretakers completed a structured electronic survey capturing self-identified sociodemographic characteristics; vaccination status; and disease symptom types, onset, and trajectory. Trained laboratory assistants performed a bilateral anterior nasal swab for COVID-19 testing using an RAT (BinaxNOW COVID-19 Ag Card; Abbott Laboratories) and a bilateral nasal swab for sequencing.^18,19^ We performed genotyping of all isolates as previously described.^20^ Full-genome sequences are available through the Global Initiative on Sharing Avian Influenza Data (GISAID) and previously described for the pre-Delta period (most being Epsilon^21^ and Alpha variants) and for the Omicron BA.1 period.^20^ During the Omicron BA.1 period, community health workers reminded people who tested positive to repeat the test 5 days after symptom onset or their initial test date to assess candidacy for shortening isolation per California Department of Public Health guidelines.^22^ Clients received a text message reminding them of this option.

### Definitions

For analysis purposes, 3 periods were defined corresponding to 3 distinct COVID-19 periods: (1) the pre-Delta period, from January 10, 2021, to May 31, 2021; (2) the Delta period, from June 1, 2021, to November 30, 2021; and (3) the Omicron BA.1 period, from December 1, 2021, to January 30, 2022. Two different analysis populations were defined.

### Statistical Analysis

First, among 18 301 symptomatic participants, we calculated the proportion with a positive RAT result and the prevalence of specific symptoms during each variant period (Omicron BA.1, pre-Delta, and Delta), stratified by RAT result. Among persons with a positive test, we evaluated whether symptom prevalence differed between the Omicron BA.1 period and the pre-Delta and Delta periods. We also assessed symptom prevalence by age and vaccination status during the Omicron BA.1 period and determined whether self-reported symptoms improved, worsened, or remained unchanged in the days following the test.

The second analysis assessed the proportion of 942 participants during the Omicron BA.1 period with a positive repeated COVID-19 RAT. This analysis was restricted to participants who had a positive RAT result on or after January 1, 2022, and had at least 1 additional RAT 2 or more days after the initial positive test. For each day from day 4 to 14 after symptom onset, or day since initial positive test if asymptomatic, we estimated the proportion of persons who remained positive by day. Participants whose second test was positive were assumed to be positive each day between the positive tests. Test positivity on days between a positive and a negative test was imputed in 3 different ways: (1) assuming a linearly decreasing probability of testing positive between 2 tests (main analysis), (2) assuming that tests would have converted to negative the day after their initial positive test (lower bound of sensitivity analysis), and (3) assuming that tests would have remained positive until the day before their repeat negative test (upper bound of sensitivity analysis). We further stratified those analyses by symptom and vaccine status. For completeness, we also report positivity over time among repeat testers without imputation of results between tests.

For both analysis populations, Fisher exact or χ^2^ tests were used as appropriate to compare proportions. Kruskal-Wallis tests were used to compare medians. We report both unadjusted *P* values and for multiple comparisons of symptom prevalence across periods, vaccination, and age strata, *P* values adjusted using the Benjamin-Hochberg method to control the false discovery rate at 5%. A 2-sided value of *P* < .05 and 95% CIs excluding 0 for differences were considered statistically significant. Adjusted *P* values were calculated in R, version 4.0.5 (R Project for Statistical Computing), and all other analyses were performed in SciPy, version 1.4.1.

## Results

A total of 63 277 people underwent testing from January 10, 2021, to January 31, 2022. The median (IQR) age of patients was 32 (21-44) years, with 12.0% younger than 12 years; 52.0% were women and 48.0% were men. Overall, test positivity was 19.2% during the pre-Delta period, 8.5% during the Delta period, and 41.6% during the Omicron BA.1 period. Among people who were tested, 18 301 participants (28.9%) reported at least 1 symptom at the time of testing; of these, 4565 (24.9%) tested positive for SARS-CoV-2. Race and ethnicity were self-reported at the time of testing registration, and this information was collected to assess the reach of our programs. Data on race and ethnicity were missing for 1294 people. Of the 17007 individuals for whom data were available, 1032 [6.1%] were Asian; 356 [2.1%] were Black/African American; 11 856 [69.7%] were Latinx/Hispanic, including American Indian from South or Central America; 2157 [12.7%] were White; and 1606 [9.4%] were other, which included American Indian or Alaska Native, Native Hawaiian or Pacific Islander, or not disclosed. During the Omicron BA.1 period, the median (IQR) age was 34.2 (18.0-46.0) years, 3803 of 7211 (52.7%) were female, 5328 of 7176 (74.2%) were Latinx, and 5711 of 7283 (78.4%) reported an annual household income of less than $50 000 (denominators reflect numbers for whom data were missing). We detected differences in who sought testing by variant period according to age, sex, and race and ethnicity (eTable 1 in the Supplement). The numbers of unvaccinated symptomatic participants decreased throughout the variant periods: 5203 of 5533 (94.0%) during the pre-Delta period, 1166 of 2283 (51.1%) and 233 of 6777 (3.4%) during the Omicron BA.1 period (eTable 1 in the Supplement).

### Symptom Profile Among People Testing Positive for COVID-19

During the Omicron BA.1 period, the most common symptoms reported by the 3032 of 7283 symptomatic persons (41.6%) who tested positive via RAT were cough (2044 [67.4%]), sore throat (1316 [43.4%]), congestion (1177 [38.8%]), and headache (1075 [35.5%]), whereas loss of smell or taste (160 [5.3%]) and diarrhea (144 [4.8%]) were least commonly reported (Table 1). During the Omicron BA.1 period, the proportion of symptomatic COVID-19–positive persons reporting cough (2044 of 3032 [67.4%]) was higher than during the pre-Delta (546 of 1065 [51.3%], *P* < .001) and Delta (281 of 468 [60.0%], *P* = .002) periods, as was the proportion with sore throat (1316 [43.4%] vs 315 [29.6%] of 1065 participants for pre-Delta, *P* < .001; and 136 of 468 participants [29.1%] for Delta, *P* < .001). In contrast, among those in the Omicron B.1 period, reports of fever (921 [30.4%] vs 369 [34.7%] of 1065 participants for pre-Delta, *P* = .01 and 172 of 468 participants [36.8%], *P* = .006, for Delta) and loss of taste or smell (183 of 1065 participants [17.2%] for pre-Delta and 160 of 3032 [5.3%] vs 96 of 468 [20.5%] participants for Delta, *P* < .001 for both) were lower (Table 1; eFigure 1 in the Supplement). Congestion was more common among symptomatic individuals who tested positive during the Omicron BA.1 period than during the pre-Delta period (1177 of 3032 [38.8%] vs 294 of 1065 [27.6%] participants, *P* < .001). The most common COVID-19 symptoms among symptomatic people testing positive during the Omicron BA.1 period (cough, sore throat, and congestion) were also common among symptomatic people who tested negative (eFigure 1 in the Supplement).

**Table 1.** Symptoms Reported Among Symptomatic People Testing Positive or Negative With a Rapid Antigen Test, by Variant Period^a^

Symptom	Overall (n = 18 301)	No. (%) of participants						*P* value (B-H corrected)		Point estimate (95% CI)	
		Pre-Delta (n = 5533)		Delta (n = 5485)		Omicron (n = 7283)					
		Positive (n = 1065)	Negative (n = 4468)	Positive (n = 468)	Negative (n = 5017)	Positive (n = 3032)	Negative (n = 4251)	Omicron vs pre-Delta among COVID-positive patients	Omicron vs Delta among COVID-positive patients	Omicron vs pre-Delta among COVID-positive patients	Omicron vs Delta among COVID-positive patients
Fever	3527 (19.3)	369 (34.7)	672 (15.0)	172 (36.8)	780 (15.6)	921 (30.4)	613 (14.4)	.01 (.02)	.006 (.01)	0.88 (0.79-0.97)	0.83 (0.73-0.94)
Cough	8885 (48.6)	546 (51.3)	1507 (33.7)	281 (60.0)	2356 (47.0)	2044 (67.4)	2151 (50.6)	<.001 (.003)	.002 (.005)	1.31 (1.23-1.40)	1.12 (1.04-1.21)
Shortness of breath	1386 (7.6)	93 (8.7)	409 (9.2)	42 (9.0)	346 (6.9)	241 (8.0)	255 (6.0)	.42 (.53)	.45 (.54)	0.91 (0.72-1.14)	0.89 (0.65-1.21)
Fatigue	3404 (18.6)	176 (16.5)	926 (20.7)	107 (22.9)	879 (17.5)	594 (19.6)	722 (17.0)	.03 (.05)	>.99 (>.99)	1.19 (1.02-1.38)	0.86 (0.71-1.03)
Myalgia	3896 (21.3)	322 (30.2)	978 (21.9)	141 (30.1)	829 (16.5)	868 (28.6)	758 (17.8)	.32 (.43)	.51 (.58)	0.95 (0.85-1.05)	0.95 (0.82-1.10)
Headache	6064 (33.1)	437 (41.0)	1693 (37.9)	167 (35.7)	1420 (28.3)	1075 (35.5)	1272 (29.9)	.001 (.003)	.92 (.96)	0.86 (0.79-0.94)	0.99 (0.87-1.13)
Loss of taste or smell	951 (5.2)	183 (17.2)	207 (4.6)	96 (20.5)	177 (3.5)	160 (5.3)	128 (3.0)	<.001 (.003)	<.001 (.003)	0.31 (0.25-0.38)	0.26 (0.20-0.32)
Sore throat	6570 (35.9)	315 (29.6)	1399 (31.3)	136 (29.1)	1821 (36.3)	1316 (43.4)	1583 (37.2)	<.001 (.003)	<.001 (.003)	1.47 (1.33-1.62)	1.49 (1.29-1.73)
Congestion	6217 (34.0)	294 (27.6)	1216 (27.2)	193 (41.2)	1866 (37.2)	1177 (38.8)	1471 (34.6)	<.001 (.003)	.32 (.43)	1.41 (1.26-1.57)	0.94 (0.84-1.06)
Nausea	1108 (6.1)	75 (7.0)	295 (6.6)	29 (6.2)	324 (6.5)	150 (5.0)	235 (5.5)	.01 (.02)	.25 (.38)	0.70 (0.54-0.92)	0.80 (0.54-1.17)
Diarrhea	1192 (6.5)	65 (6.1)	425 (9.5)	28 (6.0)	304 (6.1)	144 (4.8)	226 (5.3)	.08 (.14)	.25 (.38)	0.78 (0.59-1.03)	0.79 (0.54-1.18)
Any of the above symptoms in isolation	6796 (37.1)	219 (20.6)	1351 (30.2)	153 (32.7)	2055 (41.0)	962 (31.7)	2056 (48.4)	<.001 (.003)	.54 (.59)	1.54 (1.36-1.76)	0.97 (0.84-1.12)

Abbreviation: B-H, Benjamin-Hochberg.

^a^ The pre-Delta variant period was January 10 to May 31, 2021; the Delta period was June 1 to November 30, 2021; and the Omicron BA.1 period was December 1, 2021, to January 30, 2022.

During the Omicron BA.1 period, 144 (47.7%) of 302 symptomatic children (<12 years of age) with COVID-19 reported only 1 symptom. In comparison with children, reporting a single symptom was less common among adults aged 18 years and older (719 of 2419 participants [29.7%], *P* < .001) and adolescents (aged 12 to 17 years) (99 of 311 participants [31.8%], *P* < .001) (eTable 2 in the Supplement). Loss of taste or smell was uncommon among children (1 of 302 individuals [0.3%]) compared with adolescents (18 of 311 participants [5.8%], *P* < .001) and adults (141 of 2419 participants [5.8%], *P* < .001) (Table 2).

**Table 2.** Symptoms Reported Among Symptomatic People Testing Positive or Negative With a Rapid Antigen Test During the Omicron BA.1 Period,^a^ Stratified by Age Group

Symptoms	Overall (n = 7283)	No. (%) of participants						*P* value (B-H corrected)		Point estimate (95% CI)	
		Age ≥18 y (n = 5779)		Age 12-17 y (n = 664)		Age <12 y (n = 840)					
		Positive (n = 2419)	Negative (n = 3360)	Positive (n = 311)	Negative (n = 353	Positive (n = 302)	Negative (n = 538)	Age ≥18 y vs 12-17 y among COVID-positive patients	Age ≥18 y vs <12 y among COVID-positive patients	Age ≥18 y vs 12-17 y among COVID-positive patients	Age ≥18 y vs <12 y among COVID-positive patients
Fever	1534 (21.1)	733 (30.3)	492 (14.6)	84 (27.0)	52 (14.7)	104 (34.4)	69 (12.8)	.23 (.46)	.14 (.31)	1.12 (0.95-1.33)	0.88 (0.69-1.12)
Cough	4195 (57.6)	1626 (67.2)	1617 (48.1)	218 (70.1)	186 (52.7)	200 (66.2)	348 (64.7)	.31 (.55)	.73 (.90)	0.96 (0.88-1.04)	1.01 (0.91-1.13)
Shortness of breath	496 (6.8)	209 (8.6)	221 (6.6)	22 (7.1)	25 (7.1)	10 (3.3)	9 (1.7)	.35 (.55)	.001 (.002)	1.22 (0.66-2.28)	2.61 (1.26-5.42)
Fatigue	1316 (18.1)	538 (22.2)	664 (19.8)	33 (10.6)	33 (9.4)	23 (7.6)	25 (4.7)	<.001 (.002)	<.001 (.002)	2.10 (1.41-3.13)	2.92 (1.76-4.85)
Myalgia	1626 (22.3)	779 (32.2)	681 (20.3)	63 (20.3)	51 (14.5)	26 (8.6)	26 (4.8)	<.001 (.002)	<.001 (.002)	1.59 (1.1-2.31)	3.74 (2.44-5.74)
Headache	2347 (32.2)	898 (37.1)	1118 (33.3)	124 (39.9)	99 (28.1)	53 (17.6)	55 (10.2)	.35 (.55)	<.001 (.002)	0.93 (0.73-1.2)	2.12 (1.6-2.8)
Loss of taste or smell	288 (4.0)	141 (5.8)	112 (3.3)	18 (5.8)	12 (3.4)	1 (0.3)	4 (0.7)	.98 (.98)	<.001 (.002)	1.01 (0.14-7.17)	17.6 (2.36-131.05)
Sore throat	2899 (39.8)	1099 (45.4)	1364 (40.6)	140 (45.0)	121 (34.3)	77 (25.5)	98 (18.2)	.89 (.93)	<.001 (.002)	1.01 (0.83-1.23)	1.78 (1.42-2.24)
Congestion	2648 (36.4)	960 (39.7)	1156 (34.4)	126 (40.5)	111 (31.4)	91 (30.1)	204 (37.9)	.78 (.90)	.001 (.002)	0.98 (0.82-1.17)	1.32 (1.06-1.64)
Nausea	385 (5.3)	118 (4.9)	182 (5.4)	16 (5.1)	21 (6.0)	16 (5.3)	32 (6.0)	.84 (.92)	.75 (.90)	0.95 (0.57-1.58)	0.92 (0.47-1.81)
Diarrhea	370 (5.1)	119 (4.9)	71 (5.1)	12 (3.9)	38 (10.8)	13 (4.3)	17 (3.2)	.41 (.60)	.64 (.88)	1.27 (0.73-2.23)	1.14 (0.53-2.46)

Abbreviation: B-H, Benjamin-Hochberg.

^a^ December 1, 2021, to January 30, 2022.

We also found differences in symptoms according to vaccination status (Table 3). Among persons receiving vaccine boosters, congestion (207 of 432 participants [47.9%]) was more common, but fever (97 [22.5%]) was less common compared with both unvaccinated people (40 of 116 participants [34.5%] for congestion, *P* = .01 and 42 of 116 participants [36.2%] for fever, *P* = .003) and vaccinated people without a booster (668 of 1705 [39.2%] for congestion, *P* = .001 and 559 of 1705 [32.8%] for fever, *P* < .001) (Table 3); myalgia was also less common among people with a booster compared with vaccinated persons without a booster (115 of 432 [26.6%] vs 580 of 1705 [34.0%] participants, *P* = .01). The prevalence of other symptoms did not differ by vaccination status.

**Table 3.** Symptoms Reported Among Symptomatic People Testing Positive or Negative With a Rapid Antigen Test During the Omicron BA.1 Period,^a^ Stratified by Vaccination Status

Symptoms	Overall (n = 5424)	No. (%) of participants						*P* value (B-H corrected)		Point estimate (95% CI)	
		Unvaccinated (n = 233)		Vaccinated							
		Positive (n = 116)	Negative (n = 117)	Not boosted (n = 3817)		Boosted (n = 1374)		Boosted vs unvaccinated among COVID-positive patients	Boosted vs vaccinated not boosted among COVID-positive patients	Boosted vs unvaccinated among COVID-positive patients	Boosted vs vaccinated not boosted among COVID-positive patients
				Positive (n = 1705)	Negative (n = 2112)	Positive (n = 432)	Negative (n = 942)				
Fever	1162 (21.4)	42 (36.2)	16 (13.7)	559 (32.8)	343 (16.2)	97 (22.5)	105 (11.2)	.003 (.01)	<.001 (.01)	0.62 (0.48-0.8)	0.68 (0.57-0.83)
Cough	3060 (56.4)	71 (61.2)	53 (45.3)	1191 (69.9)	1086 (51.4)	268 (62.0)	391 (41.5)	.87 (>.99)	.002 (.01)	1.01 (0.94-1.1)	0.89 (0.82-0.96)
Shortness of breath	408 (7.5)	10 (8.6)	7 (6.0)	150 (8.8)	150 (7.1)	36 (8.3)	55 (4.8)	.92 (>.99)	.76 (.93)	0.97 (0.68-1.37)	0.95 (0.67-1.34)
Fatigue	1144 (21.1)	33 (28.5)	25 (21.4)	372 (21.8)	410 (19.4)	104 (24.1)	200 (21.2)	.33 (.50)	.31 (.50)	0.85 (0.7-1.02)	1.10 (0.91-1.33)
Myalgia	1383 (25.5)	41 (35.3)	25 (21.4)	580 (34.0)	440 (20.8)	115 (26.6)	182 (19.3)	.06 (.19)	.003 (.01)	0.75 (0.64-0.89)	0.78 (0.66-0.93)
Headache	1921 (35.4)	49 (42.2)	42 (35.9)	646 (37.9)	719 (34.0)	153 (35.4)	312 (33.1)	.18 (.40)	.34 (.50)	0.84 (0.73-0.97)	0.93 (0.81-1.08)
Loss of taste or smell	240 (4.4)	12 (10.3)	7 (6.0)	99 (5.8)	76 (3.6)	25 (5.8)	21 (2.2)	.08 (.22)	.99 (>.99)	0.56 (0.37-0.86)	1 (0.65-1.53)
Sore throat	2325 (42.9)	48 (41.4)	34 (29.1)	776 (45.5)	850 (40.3)	208 (48.2)	409 (43.4)	.20 (.40)	.33 (.50)	1.16 (1.04-1.30)	1.06 (0.95-1.18)
Congestion	2019 (37.2)	40 (34.5)	32 (27.4)	668 (39.2)	701 (33.2)	207 (47.9)	371 (39.4)	.010 (.04)	.001 (.01)	1.39 (1.24-1.56)	1.22 (1.09-1.37)
Nausea	290 (5.4)	7 (6.0)	6 (5.1)	89 (5.2)	119 (5.6)	18 (4.2)	51 (5.4)	.39 (.50)	.37 (.50)	0.69 (0.42-1.13)	0.8 (0.49-1.31)
Diarrhea	277 (5.1)	10 (8.6)	2 (1.7)	83 (4.9)	112 (5.3)	21 (4.9)	49 (5.2)	.12 (.29)	>.99 (>.99)	0.56 (0.35-0.90)	1 (0.63-1.59)

Abbreviation: B-H, Benjamin-Hochberg.

^a^ December 1, 2021, to January 30, 2022.

### Time to Improvement of COVID-19 Symptoms During the Omicron BA.1 Period

**Figure 1.**
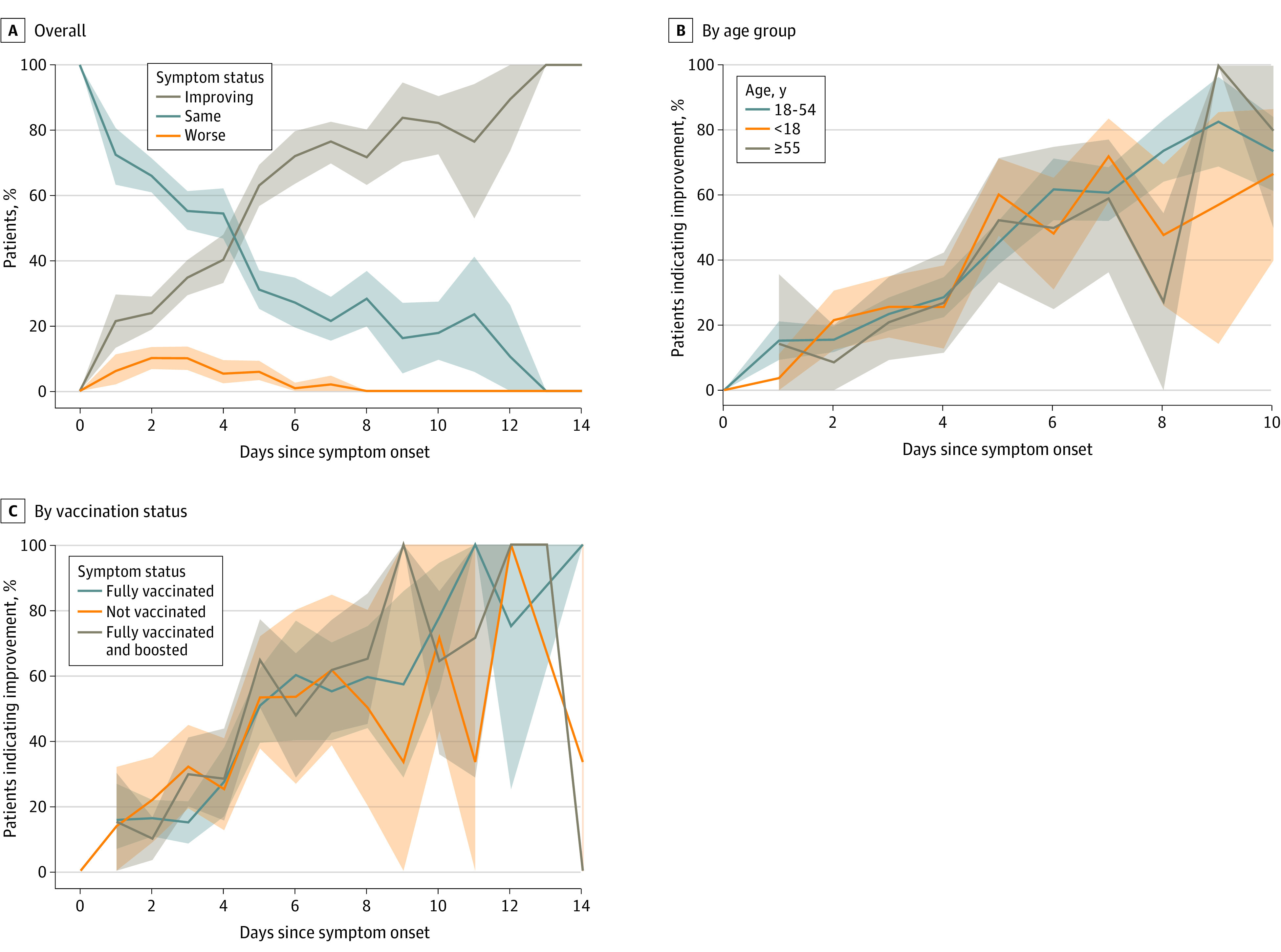
Proportion of 1613 Participants During the Omicron BA.1 Period Indicating Improvement, No Change, or Worsening of Symptoms at the Time of Testing, Among Symptomatic Rapid Antigen Diagnostic Test–Positive Participants Testing Within 14 Days of Symptom Onset, From January 7 to 31, 2022 Shaded areas indicate 95% CIs.

We evaluated the trajectory of symptoms among 1613 persons testing positive for COVID-19 during the Omicron BA.1 period (Figure 1). Among participants tested 5 days after symptom onset, 150 (63.0%; 95% CI, 56.6%-69.2%) reported that their symptoms were improving on the day of testing, 74 (31.1%; 95% CI, 25.3%-37.4%) reported similar symptoms, and 14 (5.9%; 95% CI, 3.3%-9.7%) reported worsening symptoms. Among participants testing 10 days after symptom onset, symptoms were improving in 60 (82.2%; 95% CI, 56.6%-69.2%) and were similar in 13 (17.8%; 95% CI, 9.8%-28.5%). Symptom trajectory was similar across vaccination status or age (Figure 1B and C).

### COVID-19 Repeated Testing Results

**Figure 2.**
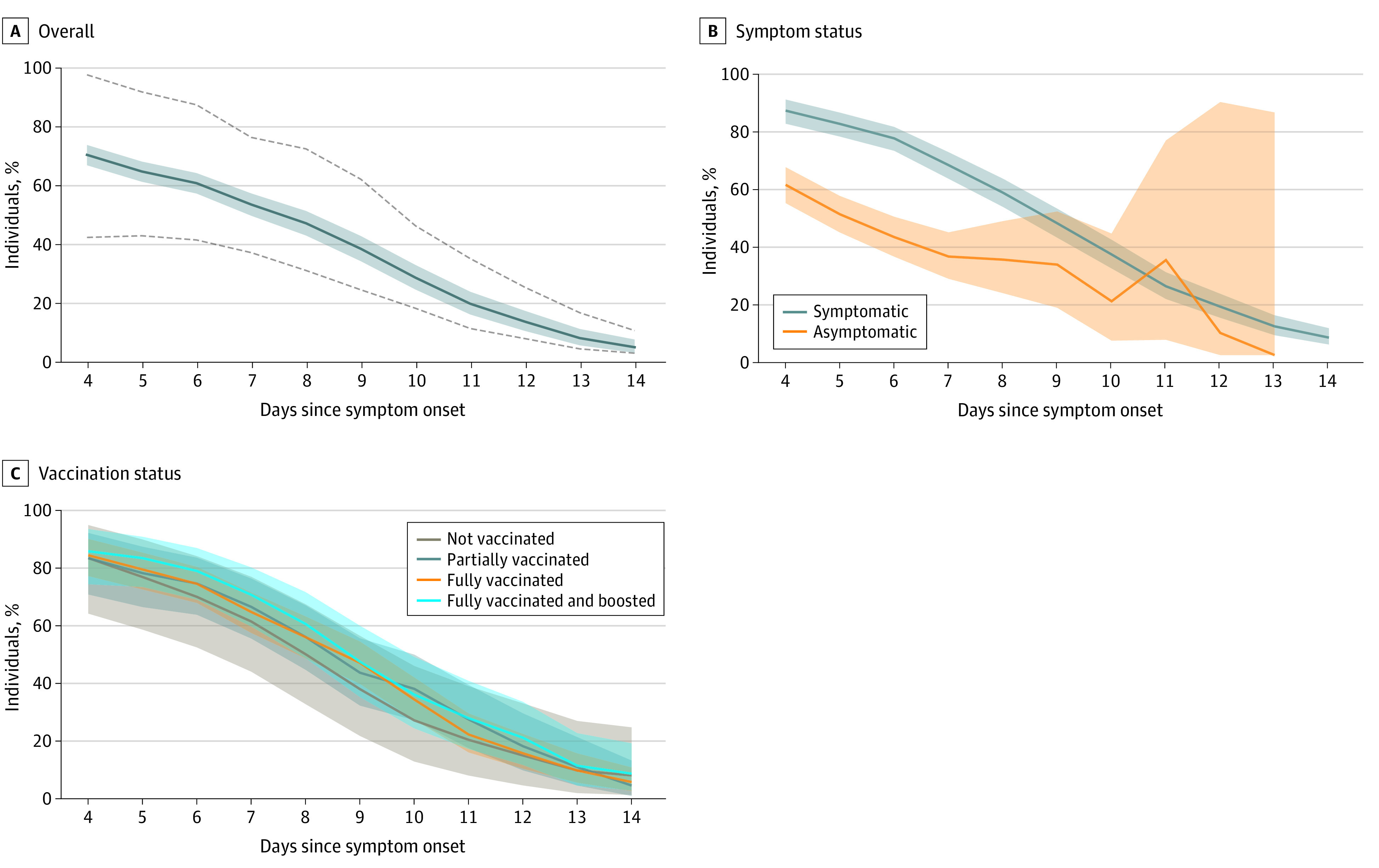
Rapid Antigen Diagnostic Test Positivity Among 942 People With COVID-19 Who Underwent Repeated Testing During the Omicron BA.1 Period by Day of Symptom Onset (if Symptomatic) or Day Since Initial Positive Test (if Asymptomatic) COVID-19 status between a participant’s tests was inferred to be positive (if second test was positive) or with a linearly decaying probability of being positive (if second test was negative). A, Results overall, including persons who were symptomatic or asymptomatic, with dotted lines indicating upper and lower bounds of sensitivity analyses. Sensitivity analyses assumed that participants whose second test was negative either became negative immediately after their first test (lower bound) or remained positive until the day before their second test (upper bound). B, Results stratified by symptom status. C, Results stratified by self-reported vaccination status as of the first rapid antigen test. Shaded areas indicate 95% CIs.

During the Omicron BA.1 period, 942 of 7832 individuals (12.0%) who tested positive by RAT were retested at the Unidos en Salud testing site. Compared with participants who did not retest (n = 6890), those who retested did not vary in age (median [IQR] age, 34.2 [21.8-44.0] years vs 32.8 [19.0-41.0] years, *P* = .25) but differed by sex (465 [49.4%] vs 3631 [52.7%] for women, *P* = .01) and by Latinx ethnicity (868 [92.1%] vs 5388 [78.2%], *P* < .001). Among 942 persons who underwent COVID-19 retesting, 65%; 95% CI, 62%-69%) remained positive via RAT 5 days after symptom onset or 5 days from their initial positive test if they were asymptomatic (Figure 2A). Among symptomatic persons, 80% (95% CI, 76%-84%) remained positive via RAT on day 5, and 35% (95% CI, 30%-40%) remained positive on day 10. Among persons asymptomatic at testing, 49% (95% CI, 43%-55%) remained positive on day 5, and 19% (95% CI, 5%-42%) remained positive on day 10. Repeated test positivity was higher among symptomatic vs asymptomatic participants (Figure 2B). We found no difference in trajectory of test positivity over time by vaccination status (Figure 2C). Similar results were found using nonimputed values for interim test positivity (eFigure 2 in the Supplement).

## Discussion

This cross-sectional study assessed the courses of 3 different COVID-19 surges, each with a different predominant SARS-CoV-2 variant and against a backdrop of rising population immunity. A shift was found toward predominantly upper respiratory tract symptoms with the Omicron BA.1 variant among symptomatic persons testing positive via RAT at a community walk-up testing site. Omicron symptom trajectories were heterogenous, with a third of people reporting no improvement or worsening of symptoms when they returned for repeated testing after 5 days. During the Omicron BA.1 period, 80.2% of symptomatic persons with COVID-19 returning for repeated testing were RAT–positive after 5 days, and 34.9% were still positive after 10 days, regardless of vaccine status. These results provide updated clinical data on outpatients with COVID-19 and RAT positivity to inform safe return to work guidelines.

Among more than 4000 symptomatic people testing positive for COVID-19 by RAT during the Omicron BA.1 surge, upper respiratory tract symptoms (sore throat and congestion) were more common than during the pre-Delta and Delta periods. The loss of taste and smell—often used by clinicians to help distinguish SARS Co-V-2 from other viral illness—was much less common during the Omicron BA.1 period (5.3%) compared with the pre-Delta (17.2%) and Delta (20.5%) periods. This shift in observed symptoms from pre-Delta to Omicron BA.1 periods is likely due to both the increase in population immunity during the Omicron BA.1 surge and the biological characteristics of this variant, which in vitro studies suggest replicates better in bronchial tissue vs deeper lung sites.^23^ During the Omicron BA.1 surge, symptoms also varied by vaccine status: congestion was more common, but fever and myalgia were less common among persons receiving a booster compared with persons who were partially vaccinated or unvaccinated.

Our findings are concordant with a population-representative household study conducted during the Omicron BA.1 period in the UK^6^ that found a predominance of upper respiratory tract symptoms. In addition, our data expand existing knowledge regarding COVID-19 symptoms by describing their differences by vaccine status and age group. Assessing the ability of symptoms to predict test positivity was beyond the scope of our analysis. However, similar to findings from the UK,^6^ we found the background rate of common COVID-19 symptoms during the Omicron BA.1 period to be high among people who tested either positive or negative. These findings emphasize the importance of ensuring that low-barrier testing is available regardless of a person’s symptom profile.

This study is one of the few in the Omicron BA.1 era to describe symptoms of mild to moderate infection in children. Although symptoms were heterogenous in children, 47.7% of children younger than 12 years had only 1 symptom, with fever, cough, and congestion being the most common. It is important to recognize that children, especially those younger than 5 years, may present with only 1 symptom. Thus, parents and health care professionals should have a low threshold for testing children with any symptom suggestive of COVID-19. Rapid and easily accessible testing is an important strategy to keep children in school while minimizing transmission.^24,25^

Despite lower frequencies of constitutional symptoms during the Omicron BA.1 surge, it is noteworthy that recovery varied, and 31.1% of patients did not report perceived improvement by day 5. Despite having illness insufficiently serious to warrant hospitalization, many individuals during the Omicron BA.1 period still felt ill on day 5 of symptoms. Safeguards routinely in place in the formal sector in the United States are often unavailable in the informal sector.^26,27^ Pandemic planning for future surges should account for these health and economic pressures.

Among persons with COVID-19 retesting on day 5, 80.2% of symptomatic people and 48.9% of asymptomatic people remained RAT–positive. The number of symptomatic participants is nearly identical to that in a community-based sample of people seeking testing in Alaska^8^ and similar to the findings in 2 cohorts of health care workers.^9,10^ This high proportion of positive repeat tests is unsurprising given existing data on the viral dynamics of Omicron BA.1 and other variants. In a cohort from the National Basketball Association composed predominantly of people who had received a booster, the duration of the acute phase of the Omicron BA.1 variant (proliferation and clearance) was 9.9 days, which is similar to prior variants.^28,29^

We found that RAT positivity remained high even 10 days following symptom onset. Additional epidemiologic studies are needed to assess whether people remain infectious at this juncture. Studies examining viral dynamics show a strong correlation between rapid antigen positivity and viable virus,^13,30,31,32^ although the correlation appears lower farther out from infection.^11,12,33^ One longitudinal study of people with the Omicron BA.1 variant found culturable virus for a median of 8 (IQR, 5-10) days,^12^ and another study from the pre-Omicron era found a strong correlation between RATs and only 1 person with culturable virus between 11 and 14 days.^11^ Overall, existing data suggest that infectiousness (using viral culture as a proxy) beyond 10 days is possible although less common.

A positive RAT result correlates with having viable virus and thus identifies persons with the highest degree of infectivity to others.^13,15,30,32^ The US Centers for Disease Control and Prevention currently allows for people leaving isolation with a well-fitting mask after 5 days of symptoms if symptoms are improving, regardless of repeated testing results.^7^ Acknowledging the need for further epidemiologic data that correlate transmission risk and RAT positivity or culturable virus, our data support current California Department of Public Health guidelines.

### Limitations

Our study has some limitations. Symptoms and timing of onset were self-reported, which may introduce bias; however, symptom data were collected prior to testing and thus would not be expected to result in differential bias between persons who tested positive or negative for COVID-19. In addition, we did not characterize the severity of COVID-19 symptoms, and even a single severe symptom may cause substantially greater morbidity in an individual than multiple mild symptoms. We did not include prior infection in this analysis and thus cannot determine the influence of symptoms or duration of test positivity. Testing was undertaken with RATs and thus may have resulted in misclassification of individuals during the earliest phases of their illness when their viral load was low. However, this should not change our overall conclusions because misclassification early in infection would result in an overestimate of the number of people with systemic symptoms. Symptomatic individuals with a negative RAT were encouraged to repeat testing in 24 to 48 hours. Finally, we noted differences in the demographic characteristics of those who retested, but to address potential bias in who retested, we stratified our analysis on variables that could be associated with duration of test positivity, such as vaccination status and symptoms. There were no data to suggest that differences by race and ethnicity or sex would be associated with the duration of RAT positivity on retesting.

## Conclusions

The clinical presentation among symptomatic persons changed with time during several COVID-19 surges that were characterized by increasing population immunity and different SARS-CoV-2 variants. During the BA.1 Omicron period, when population immunity was higher than in previous periods, persons with symptoms often did not show improvement after 5 days, and RAT results frequently remained positive. These findings highlight the importance of work assurances (ie, sick leave) to protect workers and requirements for rapid antigen testing to shorten isolation to protect the workplace. With the dynamic landscape of host immunity and viral evolution, real-time data are needed from diverse populations for clinicians, public health officials, and the community to develop optimal strategies to mitigate the health, economic, and societal effects of the SARS-CoV-2 pandemic and ensure that inequities in the treatment and outcomes of this disease are not exacerbated.

## Notes

Supplement. **eTable 1.** Demographics of Symptomatic People Seeking Testing From January 2020 to January 2021, by Variant Period **eTable 2.** Proportion of BinaxNOW Positive Symptomatic Participants Reporting Only One of the Following Symptoms at the Time of Testing During the Omicron Period (December 1, 2021-January 30, 2022) **eFigure 1.** Prevalence of Selected Symptoms Among Symptomatic People Testing Positive and Negative With the BinaxNOW Rapid Antigen Test During Pre-Delta (January 10-May 31, 2021), Delta (June 1, 2021-November 30, 2021), and the Omicron BA.1 (December 1, 2021-January 30, 2021) Periods **eFigure 2.** BinaxNOW Positivity Among 942 Repeat Testers With COVID-19 During the Omicron BA.1 Period by Day of Symptom Onset (if Symptomatic) or Day Since Initial Positive Test (if Asymptomatic)
