# Supplementary material for: COVID-19 Symptoms and Duration of Rapid Antigen Test Positivity at a Community Testing and Surveillance Site During Pre-Delta, Delta, and Omicron BA.1 Periods
Source: JAMA Netw Open. 2022 Oct 10;5(10):e2235844. doi: 10.1001/jamanetworkopen.2022.35844 (PMC9552893; doi:10.1001/jamanetworkopen.2022.35844)
Supplement: Supplement. — eTable 1. Demographics of Symptomatic People Seeking Testing From January 2020 to January 2021, by Variant Period eTable 2. Proportion of BinaxNOW Positive Symptomatic Participants Reporting Only One of the Following Symptoms at the Time of Testing During the Omicron Period (December 1, 2021-January 30, 2022) eFigure 1. Prevalence of Selected Symptoms Among Symptomatic People Testing Positive and Negative With the BinaxNOW Rapid Antigen Test During Pre-Delta (January 10-May 31, 2021), Delta (June 1, 2021-November 30, 2021), and the Omicron BA.1 (December 1, 2021-January 30, 2021) Periods eFigure 2. BinaxNOW Positivity Among 942 Repeat Testers With COVID-19 During the Omicron BA.1 Period by Day of Symptom Onset (if Symptomatic) or Day Since Initial Positive Test (if Asymptomatic) [file jamanetwopen-e2235844-s001.pdf]

Supplementary Online Content

Marquez C, Kerkhoff AD, Schrom J, et al. COVID-19 symptoms and duration of rapid antigen test positivity at a community testing and surveillance site during pre-Delta, Delta, and Omicron BA.1 periods. *JAMA Netw Open*. 2022;5(10):e2235844. doi:10.1001/jamanetworkopen.2022.35844

eTable 1. Demographics of Symptomatic People Seeking Testing From January 2020-January 2021, by Variant Period

eTable 2. Proportion of BinaxNOW Positive Symptomatic Participants Reporting Only One of the Following Symptoms at the Time of Testing During the Omicron Period (December 1, 2021-January 30, 2022)

eFigure 1. Prevalence of Selected Symptoms Among Symptomatic People Testing Positive and Negative With the BinaxNOW Rapid Antigen Test During Pre-Delta (January 10-May 31, 2021), Delta (June 1, 2021-November 30, 2021), and the Omicron BA.1 (December 1, 2021-January 30, 2021) Periods

eFigure 2. BinaxNOW Positivity Among 942 Repeat Testers With COVID-19 During the Omicron BA.1 Period by Day of Symptom Onset (if Symptomatic) or Day Since Initial Positive Test (if Asymptomatic)

This supplementary material has been provided by the authors to give readers additional information about their work.

© 2022 Marquez C et al. *JAMA Network Open*.

eTable 1: Demographics of symptomatic people seeking testing from January 2020-January 2021, by variant-period.

	**Overall (N=18,301)**	**Pre-Delta (N=5,533)**	**Delta (N=5,485)**	**Omicron (N=7,283)**	**P-value**
**Age Category (years)**					
<5	684 (3.7)	113 (2.0)	285 (5.2)	286 (3.9)	<0.001
5-11.9	1510 (8.3)	169 (3.1)	787 (14.4)	554 (7.6)	
12-17.9	1343 (7.3)	274 (5.0)	405 (7.4)	664 (9.1)	
18-30	4985 (27.2)	1725 (31.2)	1385 (25.3)	1875 (25.7)	
31-50	7020 (38.4)	2323 (42.0)	1909 (34.8)	2788 (28.3)	
51-64	2215 (12.1)	735 (13.3)	570 (10.4)	910 (12.5)	
65 and older	544 (3.0)	194 (3.5)	144 (2.6)	206 (2.8)	
**Gender**[a]					
Male	8513 (46.9)	2643 (47.8)	2517 (46.4)	3353 (46.5)	<0.001
Female	9394 (51.7)	2761 (49.9)	2830 (52.2)	3803 (52.7)	
Non-binary	234 (1.3)	106 (1.9)	73 (1.4)	55 (0.8)	
Prefer not to say	27 (0.2)	23 (0.4)	4 (0.1)	0	
**Ethnicity**[b]					
Asian	1032 (6.1)	269 (6.1)	402 (7.5)	361 (5.0)	<0.001
Black/African American	356 (2.1)	145 (3.3)	112 (2.1)	99 (1.4)	
Latinx/Hispanic	11856 (69.7)	3120 (70.2)	3408 (63.3)	5328 (74.2)	
White/Caucasian	2157 (12.7)	618 (13.9)	926 (17.2)	613 (8.5)	
Other	1606 (9.5)	295 (6.6)	536 (10.0)	775 (10.8)	
**Vaccine Status**[c]					<0.001
Not Vaccinated	6602 (45.2)	5203 (94.0)	1166 (51.1)	233 (3.4)	
Partially Vaccinated	2265 (15.5)	322 (5.8)	590 (25.8)	1353 (20.0)	
Primary Vaccine Series	4256 (29.2)	8 (0.1)	431 (18.9)	3817 (56.3)	
Primary Vaccine Series with Booster	1470 (10.1)	--[d]	96 (4.2)	1374 (20.2)	

[a]133 missing responses [b]1294 missing responses [c]3708 missing responses [d]Booster shots were not available at any point during this period [e]1657 missing responses

© 2022 Marquez C et al. *JAMA Network Open*.

eTable 2. Proportion of BinaxNOW Positive Symptomatic Participants Reporting Only One of the following Symptoms at the Time of Testing During the Omicron Period (December 1, 2021-January 30, 2022)

Symptom Type	Overall<br><br>(N=3032)	Age ≥18<br><br>(N=2419)	Age 12-17<br><br>(N=311)	Age <12<br><br>(N=302)	P-value
Fever	74 (2.4)	45 (1.9)	3 (1.0)	26 (8.6)	<0.001
Cough	477 (15.7)	346 (14.3)	48 (15.4)	83 (27.5)	0.030
Shortness of breath	4 (0.1)	4 (0.2)	0	0	1
Fatigue	12 (0.4)	10 (0.4)	0	2 (0.7)	0.41
Myalgia	25 (0.8)	22 (0.9	2 (0.6)	1 (0.3)	0.76
Headache	51 (1.7)	37 (1.5)	11 (3.5)	3 (1.0)	0.036
Loss of taste/smell	5 (0.2)	4 (0.2)	1 (0.3)	0	0.68
Sore throat	168 (5.5)	147 (6.1)	17 (5.5)	4 (1.3)	0.001
Congestion	140 (4.6)	100 (4.1)	17 (5.5)	23 (7.6)	0.019
Nausea	4 (0.1)	3 (0.1)	0	1 (0.3)	0.39
Diarrhea	2 (0.1)	1 (0.0)	0	1 (0.3)	0.20
Any of the above symptoms in isolation	962 (31.7)	719 (29.7)	99 (31.8)	144 (47.7)	<0.001

© 2022 Marquez C et al. *JAMA Network Open*.

eFigure 1. Prevalence of Selected Symptoms Among Symptomatic People Testing Positive and Negative With the BinaxNOW Rapid Antigen Test During Pre-Delta (January 10-May 31, 2021), Delta (June 1, 2021-November 30, 2021), and the Omicron BA.1 (December 1, 2021-January 30, 2021) Periods

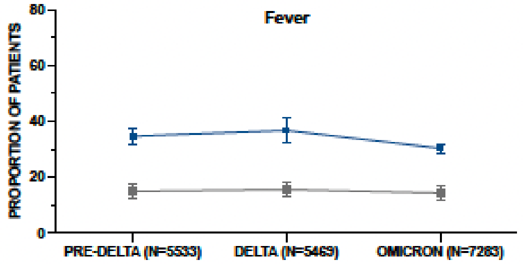

Pos.	34.7%	36.8%	30.4%
Neg.	15.0%	15.6%	14.4%

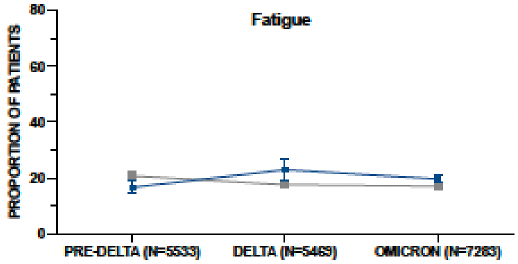

Pos.	16.5%	22.9%	19.6%
Neg.	20.7%	17.5%	17.0%

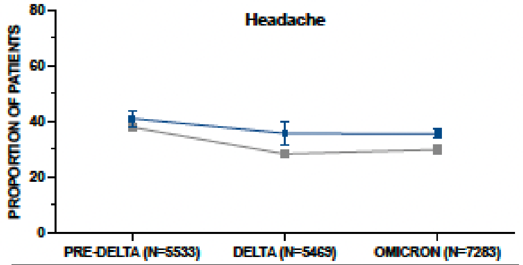

Pos.	41.0%	35.7%	35.5%
Neg.	37.9%	28.3%	29.9%

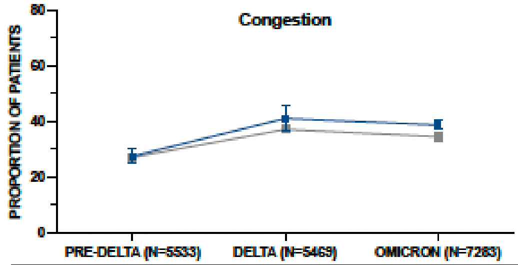

Pos.	27.6%	41.2%	38.8%
Neg.	27.2%	37.2%	34.6%

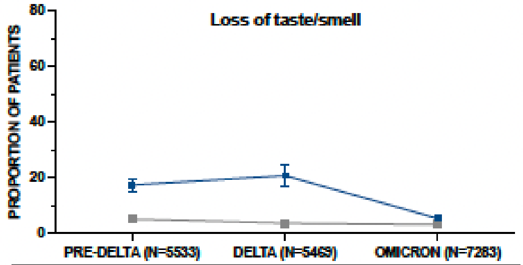

Pos.	17.2%	20.5%	5.2%
Neg.	5.6%	3.5%	3.0%

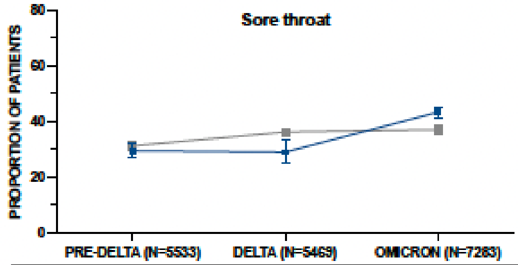

Pos.	29.6%	29.1%	43.4%
Neg.	31.3%	36.3%	37.2%

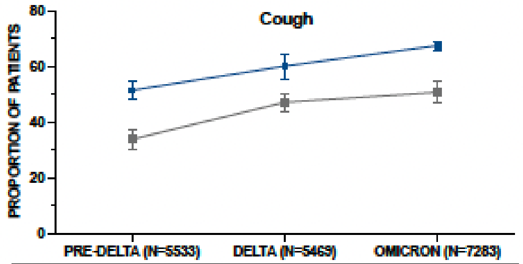

Pos.	51.3%	60.0%	30.4%
Neg.	33.7%	47.0%	50.6%

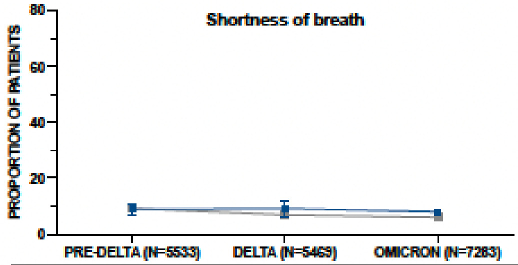

Pos.	8.7%	9.0%	7.9%
Neg.	9.2%	6.9%	6.0%

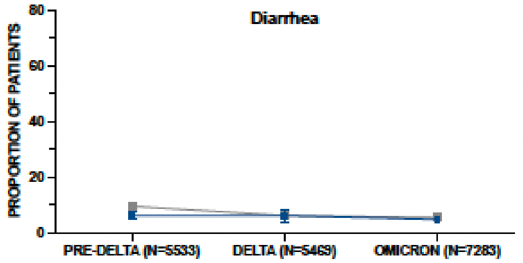

Pos.	6.1%	6.0%	4.7%
Neg.	9.5%	6.1%	5.3%

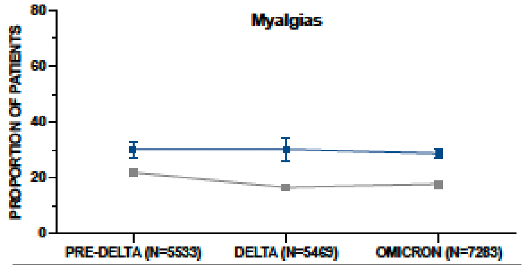

Pos.	30.2%	30.1%	28.6%
Neg.	21.9%	16.5%	17.9%

© 2022 Marquez C et al. *JAMA Network Open*.

eFigure 2. BinaxNOW Positivity Among 942 Repeat Testers With COVID-19 During the Omicron BA.1 Period by Day of Symptom Onset (if Symptomatic) or Day Since Initial Positive Test (if Asymptomatic)

A. Overall, includes symptomatic people and asymptomatic people. B. Stratified by symptom status. Symptomatic include people who report symptoms at the time of their initial positive test and who note a symptom onset date and asymptomatic people include people who do not report symptoms at the time of testing. C. Stratified by self-reported vaccination status.

© 2022 Marquez C et al. *JAMA Network Open*.

A

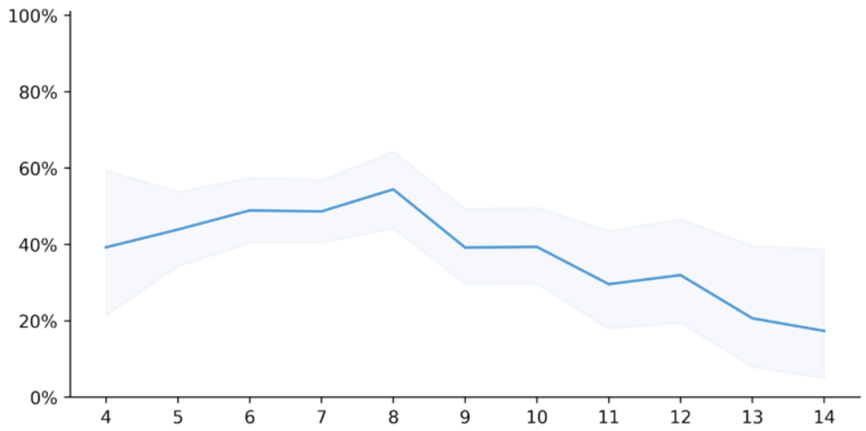

4	5	6	7	8	9	10	11	12	13	14
39.3% (11/28)	43.5% (47/107)	48.9% (69/141)	48.7% (74/152)	54.5% (55/101)	39.2% (40/102)	39.4% (39/99)	29.6% (16/54)	32.0% (16/50)	20.7% (6/29)	17.4% (4/23)

B

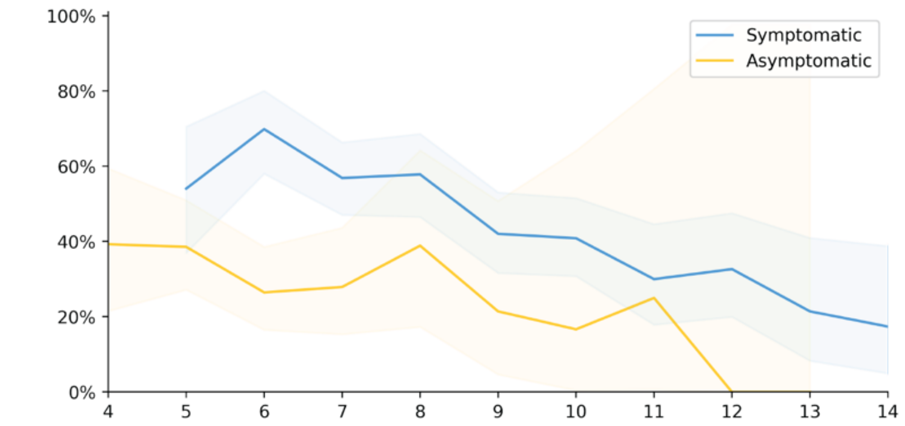

	4	5	6	7	8	9	10	11	12	13	14
Symptomatic	--	54.1% (20/37)	69.9% (51/73)	56.9% (62/109)	57.8% (48/83)	42.0% (37/88)	40.9% (38/93)	30.0% (15/50)	32.7% (16/49)	21.4% (6/28)	17.4% (4/23)
Asymptomatic	39.3% (11/28)	38.6% (27/70)	26.5% (18/68)	27.9% (12/43)	38.9% (7/18)	21.4% (3/14)	16.7% (1/6)	25.0% (1/4)	0% (0/1)	0% (0/1)	--

C

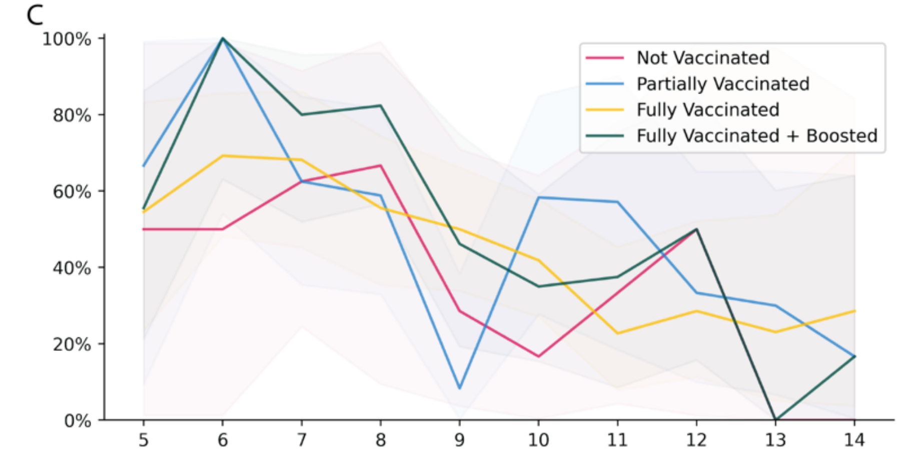

	5	6	7	8	9	10	11	12	13	14
Not Vaccinated	50.0% (1/2)	50.0% (1/2)	62.5% (5/8)	66.7% (2/3)	28.6% (2/7)	16.7% (1/6)	33.3% (2/6)	50.0% (1/2)	0.0% (0/1)	0.0% (0/2)
Partially	66.7% (2/3)	100% (6/6)	62.5% (10/16)	58.8% (10/17)	8.3% (1/12)	58.3% (7/12)	57.1% (4/7)	33.3% (4/12)	30.0% (3/10)	16.7% (1/6)
Fully	54.5% (6/11)	69.2% (18/26)	68.2% (15/22)	55.6% (15/27)	50.0% (20/40)	41.9% (18/43)	22.7% (5/22)	28.6% (6/21)	23.1% (3/13)	28.6% (2/7)
Boosted	55.6% (5/9)	100% (8/8)	80.0% (12/15)	82.4% (14/17)	46.1% (6/13)	35.0% (7/20)	37.5% (3/8)	50.0% (4/8)	0.0% (0/4)	16.7% (1/6)

© 2022 Marquez C et al. *JAMA Network Open*.
